# Surface Acoustic Wave Gyroscopic Effect in an Interdigital Transducer

**DOI:** 10.3390/s19010106

**Published:** 2018-12-29

**Authors:** Xueping Sun, Weiguo Liu, Xiuting Shao, Shun Zhou, Wen Wang, Dabin Lin

**Affiliations:** 1 School of Microelectronics, Xidian University, Xi’an 710126, China; sunxueping.1988@163.com; 2 Laboratory of Thin Film Techniques and Optical Test, Xi’an Technological University, Xi’an 710032, China; zsemail@126.com; 3 School of Information Science and Engineering, Shandong Normal University, Jinan 250358, China; shaoxiuting@126.com; 4 State Key Laboratory of Acoustics, Institute of Acoustics, Chinese Academy of Sciences, Beijing 100190, China

**Keywords:** surface acoustic wave, gyroscopic effect, interdigital transducer

## Abstract

The surface acoustic wave (SAW) gyroscopic effect in an interdigital transducer (IDT) deposited on a piezoelectric substrate is different from that in the piezoelectric substrate due to a reflection induced by IDT. In this work, an extended coupling-of-mode (COM) model including the gyroscopic effect and the reflection was developed to analyze the SAW gyroscopic effect. First, dispersion characteristics parameters of SAW were fitted according to the data derived using the finite element method (FEM). Then, variations of stop band edge frequency were calculated using the extended COM theory by integrating dispersion characteristics parameters into the COM model. We compared its results with those obtained via FEM analysis to confirm the proposed model’s validity. We found that the variation in stop band edge frequency related to gyroscope effect reached the maximum value with a zero reflectivity value. For split IDT, the sensitivity of gyroscope effect is 0.036 Hz/rad/s with a lower than 1% normalized thickness. Conversely, the value of sensitivity was almost zero for bidirectional IDT and electrode width controlled single-phase unidirectional transducer (EWC/SPUDT).

## 1. Introduction

Surface acoustic wave (SAW) gyroscopes have received increasing attraction because of their many unique properties including inherent shock robustness, small size, low cost, and long working life [1]. SAW gyroscopes can be used to measure angular rates through a rotation-induced secondary SAW or rotation-induced frequency shifts in SAW on the surface of the piezoelectric material. Its basic behaviors can be explained by the equations of a rotating piezoelectric body incorporating the rotation-related Coriolis and centrifugal accelerations [2].

Much research on the gyroscopic effect has been conducted, which has simplified it as rotation-affected vibration or wave. Huston [3] investigated the effect of rigid body rotation on wave propagation velocities in elastic media and concluded that the rotation tends to increase the velocity of one type of waves while decreasing the other. Rotation effects on wave speed in isotropic elastic surface wave resonators has been analyzed [4,5,6,7,8]. Chen [8] introduced the gyroscopic gain factor, which represents the relationship between the velocity variation and the frequency variation according to the applied angular rate. Fang et al. [9,10] analyzed the surface acoustic waves propagating over a piezoelectric half-space rotating at a constant angular velocity. An analysis of the effect of rotation on SAW propagation was presented in a piezoelectric half-space [11]. The results showed that the velocity varies in the Rayleigh wave and the Bleustein–Gulyaev (BG) wave with the ratio of angular rate to the frequency. Some numerical results for 128°YX-cut LiNbO_3_ and X-112°Y LiTaO_3_ were presented [1,12] to illustrate the effects of the Coriolis force and the centrifugal force on the Rayleigh wave. Sizov et al. proposed an analytical solution to the problem of elastic waves propagation in a structure comprising a carrying foundation upon which a piezoelectric layer was applied and was being subjected to constant rotation [13]. The governing equations of a linear, homogeneous, and transversely isotropic rotating micropolar piezoelectric medium for surface wave solutions were solved by Singh and Sindhu [14].

For the SAW gyroscopes reported [15,16,17,18,19,20,21,22,23,24,25,26,27,28,29], the gyroscopic effect of the following three elements need to be considered: (1) the interdigital transducer (IDT), whose type is the most important for realizing low insertion loss and improving the stability of the device; (2) the metallic dot array, which is used to enhance the Coriolis force; and (3) the semi-infinite piezoelectric substrate. The gyroscope effects of the following three type IDTs were compared experimentally: a general bidirectional IDT, split IDT, and an electrode width controlled single-phase unidirectional transducer (EWC/SPUDT) with combed IDT. The results showed that the gyroscope effect in SPUDT with combed structure is strongest [23]. Based on the experimental result, we theoretically analyzed the gyroscope effect for the commonly used IDT.

The reflection of surface acoustic waves from metal strips or a metallic dot array is an important phenomenon. The reflection affects the incident wave and causes velocity dispersion. The analysis of the excitation and the effects of periodic perturbation by IDTs is complex and an exact analysis is difficult unless finite element method (FEM) or other suitable tools are used. The coupling-of-mode (COM) theory can be used to approximate the stop band property with considering the effects of reflection and can reasonably analyze the propagation in IDTs. However, in general COM theory, the wave numbers of the two counterpropagating SAW, which have a strong coupling, are the same. Some studies indicate that the gyroscopic effect causes right and left moving waves to have different wave speeds [5,6]. Until now, there has been no discussion about the gyroscopic effect in IDTs and metal dots. 

A new extended COM theory considering the velocity dispersion caused by the SAW gyroscope effect is presented in this paper. This method can be used for effectively analyzing the frequency shift, which is a metric of the SAW gyroscopic effect, by calculating the difference between the upper stop band frequency and the lower stop band frequency. The model provides effective guidance for the structure design of SAW gyroscopes. Finally, the validity of the theory is verified by comparing its analysis results with those of the FEM method. 

## 2. Theoretical Model

IDTs are composed of periodic metal strips that disturb the propagating surface acoustic waves via short-circuiting the piezoelectric field, mass loading, and elastic stress loading [30]. Although COM theory is a phenomenological model, it is the most adequate for the phenomena being considered: excitation, propagation, and scattering of surface acoustic waves. COM simultaneously avoids making too strict hypotheses about the wave behavior on each electrode; therefore, this model is widely applied to the analysis of SAW devices.

The general COM equations in the periodic structure are:

(1) $$\begin{array}{l} \frac{\partial u_{+}}{\partial x}=-j\beta u_{+}-j\kappa_{12}u_{-}\exp\left(-j\frac{2\pi }{p}x\right)\\ \frac{\partial u_{-}}{\partial x}=j\kappa_{12}^{*}u_{+}\exp\left(j\frac{2\pi }{p}x\right)+j\beta u_{-} \end{array}$$

where *u_±_* is the mode amplitude propagating toward the *±x* direction and *κ*_12_ is the reflection coefficient.

Considering the wave number propagation on the *x*-axis is not the same as in the −*x* direction, the COM equations become:

(2) $$\begin{array}{l} \frac{\partial u_{+}}{\partial x}=-jk_{+}u_{+}-j\kappa_{12}u_{-}\exp\left(-j\frac{2\pi }{p}x\right)\\ \frac{\partial u_{-}}{\partial x}=j\kappa_{21}u_{+}\exp\left(j\frac{2\pi }{p}x\right)+jk_{-}u_{-} \end{array}$$

For the existence of nontrivial solutions, we obtain the dispersion equation that includes the gyroscopic effect.

(3) $$\left(k_{+}-\frac{\pi }{p}\right)\left(k_{-}-\frac{\pi }{p}\right)-\kappa_{12}\kappa_{21}=0$$

Through introducing the velocity dispersion obtained in Section 3.2 into the relationship *k* = *ω/v*, which requires only the first two terms. The wave number dispersions can be written as:

(4) $$\begin{array}{l} k_{+}=\frac{\omega }{v_{-}}=\frac{\omega }{v_{0}\left(1-\frac{a}{v_{0}}\left(\frac{\Omega }{\omega }\right)+\frac{b}{v_{0}}{\left(\frac{\Omega }{\omega }\right)}^{2}\right)}\approx \frac{\omega }{v_{0}}\left(1+\frac{a}{v_{0}}\left(\frac{\Omega }{\omega }\right)+\left({\left(\frac{a}{v_{0}}\right)}^{2}-\frac{b}{v_{0}}\right){\left(\frac{\Omega }{\omega }\right)}^{2}\right)\\ k_{-}=\frac{\omega }{v_{+}}=\frac{\omega }{v_{0}\left(1+\frac{a}{v_{0}}\left(\frac{\Omega }{\omega }\right)+\frac{b}{v_{0}}{\left(\frac{\Omega }{\omega }\right)}^{2}\right)}\approx \frac{\omega }{v_{0}}\left(1-\frac{a}{v_{0}}\left(\frac{\Omega }{\omega }\right)+\left({\left(\frac{a}{v_{0}}\right)}^{2}-\frac{b}{v_{0}}\right){\left(\frac{\Omega }{\omega }\right)}^{2}\right) \end{array}$$

The dispersion Equation (3) may have two solutions generally, which can be expressed as Equation (5):

(5) $$\begin{array}{l} \frac{\omega_{+}}{v_{0}}=\frac{\pi }{p}+\left({\left(\frac{a}{v_{0}}\right)}^{2}-\frac{b}{v_{0}}\right)\frac{\Omega^{2}}{\omega v_{0}}+\sqrt{{\left(\frac{a}{v_{0}^{2}}\right)}^{2}\Omega^{2}+\kappa^{2}}\\ \frac{\omega_{-}}{v_{0}}=\frac{\pi }{p}+\left({\left(\frac{a}{v_{0}}\right)}^{2}-\frac{b}{v_{0}}\right)\frac{\Omega^{2}}{\omega v_{0}}-\sqrt{{\left(\frac{a}{v_{0}^{2}}\right)}^{2}\Omega^{2}+\kappa^{2}} \end{array}$$

From Equation (5), we calculated the frequency shifts of the stop band edges due to the rotation in the existence of the reflection. 

For the upper stop band edge:

(6) $$\Delta f_{+}=\frac{1}{2\pi }\left[\left({\left(\frac{a}{v_{0}}\right)}^{2}-\frac{b}{v_{0}}\right)\frac{\Omega^{2}}{\omega }+v_{0}\left(\sqrt{{\left(\frac{a}{v_{0}^{2}}\right)}^{2}\Omega^{2}+\kappa^{2}}-\kappa \right)\right]$$

For the lower stop band edge:

(7) $$\Delta f_{-}=\frac{1}{2\pi }\left[\left({\left(\frac{a}{v_{0}}\right)}^{2}-\frac{b}{v_{0}}\right)\frac{\Omega^{2}}{\omega }-v_{0}\left(\sqrt{{\left(\frac{a}{v_{0}^{2}}\right)}^{2}\Omega^{2}+\kappa^{2}}-\kappa \right)\right]$$

A parameter Δ*f* was defined to represent the SAW gyroscopic effect:

(8) $$\Delta f=\Delta f_{+}-\Delta f_{-}=\frac{v_{0}}{\pi }\left(\sqrt{{\left(\frac{a}{v_{0}^{2}}\right)}^{2}\Omega^{2}+\kappa^{2}}-\kappa \right)$$

From Equation (8), it is clear that the reflectivity considerably influences the gyroscopic effect.

## 3. SAW in Rotating Media

### 3.1. SAW in a Rotation Semi-Infinite Piezoelectric Material

**Figure 1:**
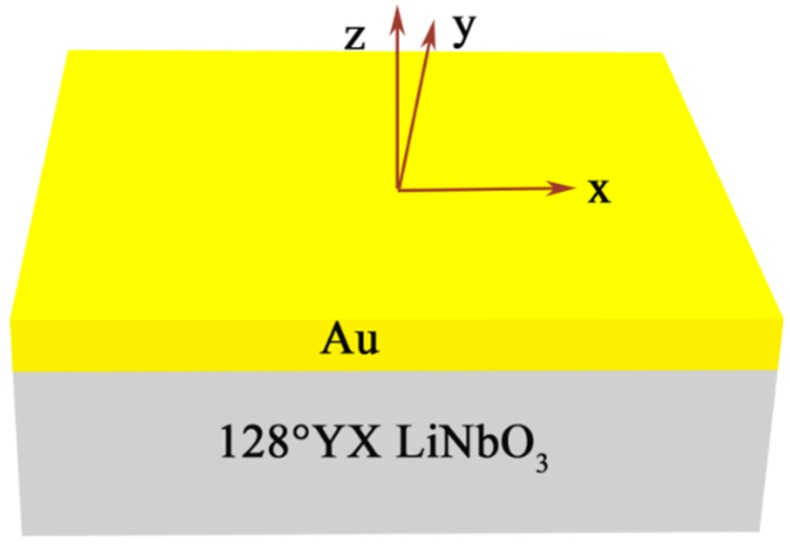
The layered structure and its coordinate system.

**Figure 2:**
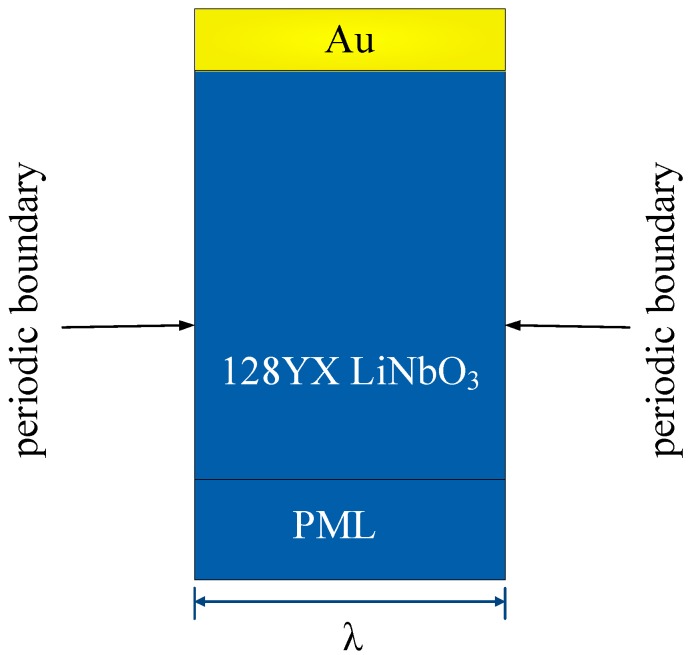
Two-dimensional (2D) periodic finite element method (FEM) model without Al electrodes used in the simulation.

The partial-wave analysis method [1,22] was used to consider the SAW gyroscope effect in a structure composed of a semi-infinite piezoelectric substrate and an Au layer over the substrate. The structure is shown in Figure 1. According to the partial-wave analysis method, the SAW velocity shift, depending on the ratio of the rotation rate by the wave frequency Ω/*ω*, can be calculated. FEM could also be used to analyze the SAW gyroscopic effect of the structure. The results were verified with the partial-wave analysis method. A two-dimensional FEM model was established as shown in Figure 2. 

In this model, the thicknesses of semi-infinite substrates are replaced by a finite thickness 5*λ* and a perfectly matched layer (PML) with a thickness of 1*λ*. The thickness of Au layer was set to 0 nm, 300 nm, 600 nm, and 900 nm. The PML can gradually absorb the mechanical and electrical disturbances in the layer before they reach the outer boundaries [31,32]. This induces a decrease in model size. Eigen frequency simulations were completed to evaluate the characteristics of propagation with the following conditions: (1) the left and right boundaries are periodic boundaries and (2) a rotating frame is applied with a given angular velocity.

According to the calculation, the SAW eigenfrequencies of a desired mode can be obtained. The upper stop band edges *f_sc+_* and the lower stop band edge *f_sc-_* are the same when the given angular velocity is zero due to the lack of reflection. When the angular velocity is not zero, the upper and lower stop band edges will split. The SAW phase velocity can be calculated using the relation:

(9) $$v_{p}=f_{sc\pm }\lambda$$

**Figure 3:**
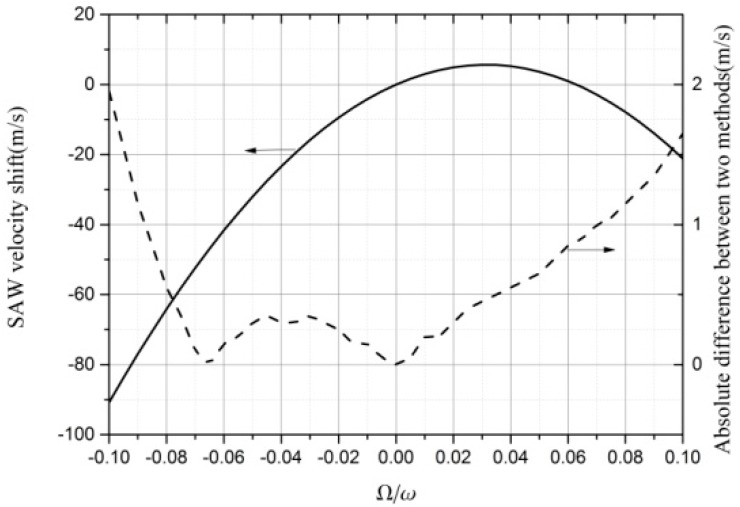
SAW velocity shift and the difference between the methods of FEM and partial–wave analysis method depending on the ratio of the rotation rate by the wave frequency Ω/*ω*.

The results of the SAW velocity shift process without an Au layer are shown in the left of Figure 3. The difference in the results between the two methods (FEM and partial–wave analysis method) are shown in the right of Figure 3. 

The results obtained by FEM were accurate, with a maximum difference not larger than 2 m/s when |Ω/*ω*| ≤ 0.1. The smaller the |Ω/*ω*|, the smaller the difference. When setting the direction of the propagation as *x*, the results of the velocity shift are shown in the curve on the left part of Figure 3. When setting the direction of the propagation to −*x*, the other curve was produced. The two curves are symmetric to |Ω/*ω*| = 0, therefore the results propagated in the −*x* axis are not shown in Figure 3. We considered that the SAW velocity shift propagation along the x-direction is |Ω/*ω*| ≥ 0.1 in Figure 3, whereas the SAW velocity shift along the −*x*-direction is |Ω/*ω*| < 0. The velocity can be approximated as a quadratic function. The fitting result of 128°YX-cut LiNbO_3_ propagation along the *x*-axis and along the −*x*-axis are:

(10) $$\begin{array}{l} V_{+}=V_{0}+a\left(\frac{\Omega }{\omega }\right)-b{\left(\frac{\Omega }{\omega }\right)}^{2}=3983.3+352.2\left(\frac{\Omega }{\omega }\right)-5603.6{\left(\frac{\Omega }{\omega }\right)}^{2}\\ V_{-}=V_{0}-a\left(\frac{\Omega }{\omega }\right)-b{\left(\frac{\Omega }{\omega }\right)}^{2}=3983.3-352.2\left(\frac{\Omega }{\omega }\right)-5603.6{\left(\frac{\Omega }{\omega }\right)}^{2} \end{array}$$

**Figure 4:**
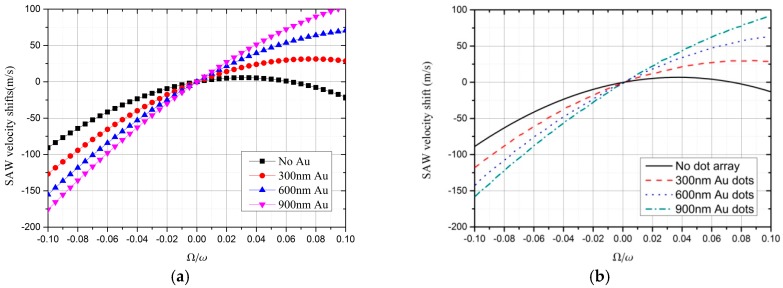
SAW velocity shift depending on Ω/*ω* under different Au thicknesses calculated by (**a**) FEM and (**b**) partial-wave method [1,14].

The relationships between the SAW velocity shift and Ω/*ω*, when different thicknesses of Au coating are deposited on the piezoelectric substrate, are shown in Figure 4. Figure 4a displays the results obtained by FEM, while Figure 4b depicts the results obtained via partial-wave analysis. The analysis of the results were discussed by Wang et al. [1,12]. We want to elaborate upon the parameters *a*, *b*, and *v*_0_. From Figure 4a, the curve is not the same when the thickness of Au layer is different; therefore, parameters *a*, *b*, and *v*_0_ change with Au thickness. 

### 3.2. SAW in Rotating IDTs

**Figure 5:**
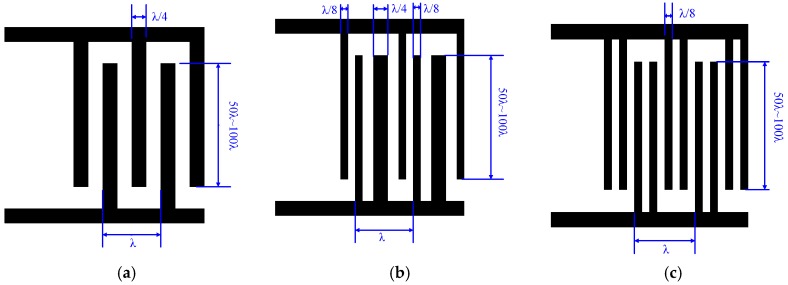
Three different interdigital transducer (IDT) structures: (**a**) bidirectional IDT, (**b**) electrode width controlled single-phase unidirectional transducer (EWC/SPUDT) IDT, and (**c**) split IDT.

The structures of three different IDT types, bidirectional IDT, EWC/SPUDT, and split IDT, which are commonly used in SAW devices, are shown in Figure 5.

**Figure 6:**
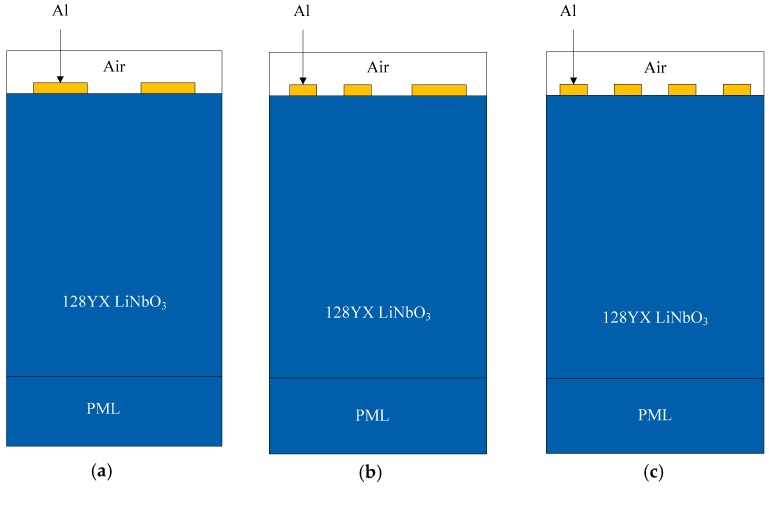
FEM models for the three types IDTs: (**a**) bidirectional IDT, (**b**) EWC/SPUDT, and (**c**) split IDT.

We investigated the influence of the SAW reflection characteristics of IDT distributed on 128°YX LiNbO_3_ substrate on the SAW gyroscope effect. The main wave mode in 128°YX LiNbO_3_ is a Rayleigh wave, which means that the particle vibration displacement is mainly in a two-dimensional plane. So, the whole structure was simplified into a two-dimensional (2D) model. As shown in Figure 6, the 2D FEM models in this section are three types of IDT on 128°YX LiNbO_3_ substrate, and Al was chosen as the electrode material for simulations, which is the same choice as Oh et al. [23].

The thickness of Al is normalized by the SAW wavelength *λ*, which equals the IDT period. The thickness of 128°YX-LiNbO_3_ and PML were 5*λ* and 1*λ*, respectively. The other parameters were the same as in Section 3.1. The velocity $v_{p}$ of a Rayleigh wave and the reflectivity $\kappa_{p}$ can be calculated using the following equations [33,34,35]:

(11) $$v_{p}=\frac{\left(f_{sc+}+f_{sc-}\right)\lambda }{2}$$

(12) $$\kappa_{p}=2\pi \frac{f_{sc+}-f_{sc-}}{f_{sc+}+f_{sc-}}$$

**Figure 7:**
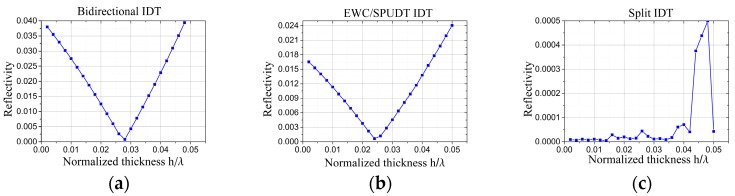
Reflectivity in different types IDTs: (**a**) bidirectional IDT, (**b**) EWC/SPUDT, and (**c**) split IDT.

The calculated reflectivity of the three structures are shown in Figure 7. These results indicate that the reflectivity reduces to zero at first, and then increases with *h*/*λ* for bidirectional IDTs and EWC/SPUDT. The reflectivity of the split IDT maintains a small value in a wide range of *h*/*λ*. Specifically, zero reflectivity appears at the point of *h*/*λ* = 2.8% in bidirectional IDTs, which is consistent with the experimental results [36]. This phenomenon occurs because the sign of the electrical reflection induced by the short-circuited grating is opposite to that of the mechanical reflection. The electrical reflection could even drop to zero at a specific thickness (such as *h*/*λ* = 2.8% in Figure 7a.

**Figure 8:**
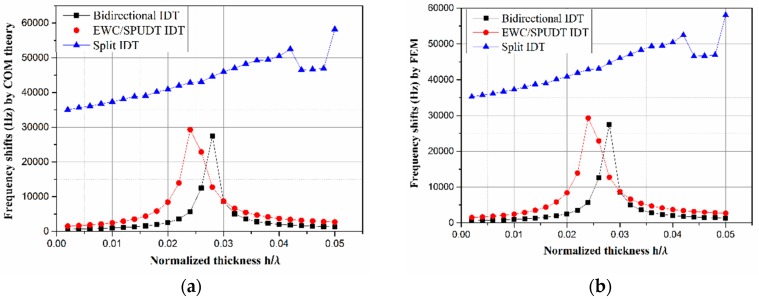
Frequency shifts for three types of IDTs (**a**) calculated by the extended coupling-of-mode (COM) theory and (**b**) by FEM.

The frequency shifts Δf reflect the intensity of the gyroscopic effect. The eigenfrequencies can be simulated directly by FEM when the angular rate is set to zero and 10^6^ rad/s. Then, the frequency shifts can be obtained by processing theses eigenfrequencies. Figure 8 shows the corresponding simulation results. The results do not explain the internal function and mechanism of the metal electrodes clearly using FEM method. Therefore, it was necessary to introduce our extended COM theory to help us understand its physical meanings.

To apply the extended COM theory, three additional parameters—*a*, *b*, and *v*_0_ in Equations (6)–(8), respectively—had to be obtained. An effective approach was employed to fit their variation with the thickness of the IDT by least-squares regression after determining the eigenfrequencies. The fitting results of the three different structures are shown in Table 1.

Next, Δf can be calculated using Equation (8). We observed that the results obtained by the extended COM theory, shown in Figure 8a, are in good agreement with those obtained by FEM shown in Figure 8b. Compared with the semi-infinite structure without IDTs, reflection occurred in the structure with IDTs. When the reflectivity approached zero, $\kappa {\left(\frac{a}{v_{0}^{2}}\Omega \right)}^{-1}$ was so small that Equation (8) can be approximated with the following form:

(13) Δf=1πav0Ω(1−κaΩv02)

Conversely, when the reflectivity is large, $\frac{a}{v_{0}^{2}}\Omega \kappa^{-1}$ was small and Equation (8) becomes:

(14) Δf=1πav0ΩaΩv02κ

Substituting the parameters *a*, *b*, and *v*_0_ in Table 1 into Equations (13) and (14), we found that the frequency shift with a zero reflectivity was nearly $\kappa {\left(\frac{a}{v_{0}^{2}}\Omega \right)}^{-1}$ times larger than that with a large reflectivity. These two limiting cases correspond to the split IDT and bidirectional IDT with thin electrodes, respectively. These analyses preliminarily proved the theory that a smaller reflectivity is obtained with a greater frequency of shift.

Considering both Figure 7 and Figure 8, the hypothesis is further confirmed. For example, the frequency shifts in the bidirectional IDT and EWC/SPUDT shown in Figure 8a increases with the decrease in the reflectivity in Figure 7a,b, respectively, until the reflectivity drops to its lowest point when *h*/*λ* = 2.8% in Figure 6a or *h*/*λ* = 2.4% in Figure 6b. At this point, the frequency shift reaches the maximum at 27,484.7 Hz and 29,294.6 Hz, respectively, which means the strongest SAW gyroscopic effect was obtained. Although the frequency shifts in bidirectional IDTs and EWC/SPUDT produced similar relation curves with their reflectivity, the frequency shift of EWC/SPUDT was larger due to its lower reflectivity when *h*/*λ* was set to the same value. For example, with an Al thickness of 200 nm (*λ* = 50 μm), the reflectivity of the EWC/SPUDT was 1.53% and its frequency shift reached 1665 Hz, whereas the values for the bidirectional IDTs were 3.55% and 698.1 Hz, respectively, as shown in Table 2. The frequency shift of the split IDTs was much larger than that of the bidirectional IDT and EWC/SPUDT IDT. This is because when *h*/*λ* is in the range of 0 to 0.05, its reflectivity, always a number close to 0, is much smaller than that of the other two structures. This frequency shift corresponds to a sensitivity of 0.036 Hz/rad/s, whereas an almost zero sensitivity exists in bidirectional and EWC/SPUDT IDT.

Notably, when the angular rate was constant, the maximum frequency shift in piezoelectric substrate distributed IDTs was slightly larger than that in semi-infinite piezoelectric substrate. This is due to the introduction of the mass loading effect by the interdigital electrodes, which leads to the slight decrease in the phase velocity while the center frequency decreases slightly as well, resulting in a slight increase in sensitivity.

All the above analyses show that lower reflections lead to higher sensitivity. In terms of experiments, several valuable references are provided by Oh et al. [23]. Their experimental results indicated that the SAW gyroscope with split IDT demonstrates higher sensitivity than that with bidirectional IDT, which is consistent with our theoretical analysis results. With regard to the gyroscope with EWC/SPUDT, a few EWC/SPUDT and many combed fingers are arranged alternately, in which the combed fingers can be equivalent to the split IDTs with all electrodes grounded. This reduces the reflectivity of the whole IDT, which is conducive to producing a higher SAW gyroscopic effect. In addition, a unidirectional SAW is generated by EWC/SPUDT, which means more acoustic energy is coupled with metal dot arrays distributed on the SAW propagation path than in a bidirectional SAW. This further enhances the strength of the SAW gyroscopic effect. Therefore, the structure of EWC/SPUDT with massive combed fingers is the most sensitive. This conclusion shows that this is an effective method to improve the detection sensitivity of SAW gyroscopes through optimizing the structure of the gyroscope based on reducing its reflectivity. 

## 4. Conclusions

An extended COM model was proposed in this study to explain the gyroscopic effect in IDT. Frequency shifts for three types IDTs were calculated using the extended COM theory, and the relevant results agreed well with those obtained directly by FEM. When the angular rate was set to 10^6^ rad/s, the frequency shifts in the bidirectional IDT and EWC/SPUDT reached their maximum of 27,484.7 Hz and 29,294.6 Hz at *h*/*λ* = 2.8% and *h*/*λ* = 2.4%, respectively. The frequency shift in the split IDT was always higher than in other two IDTs due to a low reflectivity when *h*/*λ* ranged from 0 to 0.05. When the Al electrodes had the same thickness of 200 nm in the three types IDTs, their frequency shifts were 698.1 Hz in bidirectional IDT, 1665.0 Hz in EWC/SPUDT, and 35,718.3 Hz in split IDT, and their corresponding reflectivities were 3.55%, 1.53%, and 0. According to these results, we conclude that a lower reflection leads to a larger frequency shift, which means a stronger gyroscopic effect. Therefore, reducing the reflectivity in IDT effectively improved the detection sensitivity of SAW gyroscopes. 

## Figures and Tables

**Table 1:** Values of dispersion parameters *a*, *b*, and *v*_0_.

	*a*			*b*			*v*_0_		
	(*h*/*λ*)^0^	(*h*/*λ*)^1^	(*h*/*λ*)^2^	(*h*/*λ*)^0^	(*h*/*λ*)^1^	(*h*/*λ*)^2^	(*h*/*λ*)^0^	(*h*/*λ*)^1^	(*h*/*λ*)^2^
Bidirectional IDTs	418.7	2540	34,870	5452	−2680	−72,030	3901	−823.8	−19,600
EWC/SPUDT	423.2	2431	49,870	5428	−3005	−86,810	3894	−983.4	−20,990
Split IDTs	425.3	2660	57,570	5414	−3660	−93,180	3890	−1163.0	−22,910

**Table 2:** Reflectivity and frequency shift of the three different IDTs.

IDT Type	*κ_p_*	Frequency Shift (Hz)
Bidirectional IDTs	3.55%	698.1
EWC/SPUDT	1.53%	1665.0
Split IDTs	6.6 × 10^−6^	35,718.3
